# Beyond the Biological Effect of a Chemically Characterized Poplar Propolis: Antibacterial and Antiviral Activity and Comparison with Flurbiprofen in Cytokines Release by LPS-Stimulated Human Mononuclear Cells

**DOI:** 10.3390/biomedicines7040073

**Published:** 2019-09-21

**Authors:** Paolo Governa, Maria Grazia Cusi, Vittoria Borgonetti, José Mauricio Sforcin, Chiara Terrosi, Giulia Baini, Elisabetta Miraldi, Marco Biagi

**Affiliations:** 1 Department of Biotechnology, Chemistry and Pharmacy—Department of Excellence 2018-2022, University of Siena, 53100 Siena, Italy; paolo.governa@unisi.it; 2 Department of Medical Biotechnologies, Microbiology Unit, University of Siena, 53100 Siena, Italychiara.terrosi@unisi.it (C.T.); 3 Department of Neuroscience, Psychology, Pharmacology and Child Health, University of Florence, 50139 Florence, Italy; vittoria.borgonetti@unifi.it; 4 Institute of Biosciences, São Paulo State University, Botucatu 18618-970, Brazil; jose.m.sforcin@unesp.br; 5 Department of Physical Sciences, Earth and Environment, University of Siena, 53100 Siena, Italy; baiocca89@gmail.com (G.B.); elisabetta.miraldi@unisi.it (E.M.)

**Keywords:** poplar propolis, anti-inflammatory, cytokines release, flurbiprofen, flavonoids, antibacterial, influenza virus, neuraminidase

## Abstract

Bee propolis, especially Euro-Asian poplar propolis, is among the most well-known natural products traditionally used to treat pharyngitis and minor wounds. The aim of this research was to investigate the pharmacological properties responsible for poplar propolis effectiveness using, for the first time, different in vitro approaches applied to a chemically characterized sample. The anti-inflammatory activity was compared with flurbiprofen by determining pro-inflammatory cytokines released by lipopolysaccharide-stimulated human peripheral blood mononuclear cells (PBMC). The antibacterial activity against Gram+ and Gram- bacteria was assessed, as well as antiviral effects on H1N1 influenza a virus. Poplar propolis (5 and 25 µg/mL) exerted a concentration-dependent anti-inflammatory activity. In this range of concentrations, propolis effect was not inferior to flurbiprofen on cytokines released by lipopolysaccharide (LPS)-stimulated human PBMC. Poplar propolis was found to upregulate IL-6 and IL-1β in non-stimulated PBMC. *S. aureus*, *S. pyogenes*, and *S. pneumoniae* were the most susceptible bacterial strains with inhibitory concentrations ranging from 156 to 625 µg/mL. A direct anti-influenza activity was not clearly seen. Effective anti-inflammatory concentrations of propolis were significantly lower than the antibacterial and antiviral ones and results suggested that the anti-inflammatory activity was the most important feature of poplar propolis linked to its rationale use in medicine.

## 1. Introduction

Propolis is one of the most precious products produced by bees and mankind has learned to appreciate and use it since immemorial times. It is a resinous product made by honeybees: they first collect the resins from gems, buds, and exudates of the plants and mix with bee enzymes, wax, and pollen to obtain a malleable and compact product [1]. Propolis is used by bees to repair the combs and to protect the hives, preventing microbial infection of larvae [2]. The chemical and physical characteristics of propolis, such as color, smell, taste, and consistency, are dramatically dependent upon the vegetal origin and the geographical region where it was produced. The Euro-Asian propolis, mainly derived from *Populus* spp., contains many simple phenolic acids [3]. Flavonoids represent the predominant phenolic constituents in numerous samples of different origin. Flavones, flavanones, flavonols, isoflavones, and isoflavans are present in various types of propolis, which differ in quality and abundance of such compounds. Galangin, luteolin, rhamnetin, isorhamnetin, kaempferol, quercetin and its dimethoxy derivatives are the most common flavonols in European and Asian poplar type propolis. Pinocembrin is a peculiar flavanone found in many different propolis [3]. Caffeic acid phenethyl ester (CAPE) is another active component present in propolis produced by European, Asian, and American honeybees [4]. The monoterpenic and sesquiterpenic compounds are responsible for the characteristic resinous odor of propolis. Tetracyclic and pentacyclic triterpenes also occur [3].

Propolis and some isolated constituents exert several pharmacological properties, such as immunomodulatory, anti-inflammatory, antioxidant, antibacterial, antiviral, antifungal, wound healing, analgesic, anti-ulcerous, among others [5,6,7]. The clinical literature, still limited, proposes the use of propolis-based products particularly for local inflammatory conditions and viral and bacterial infections (mainly for upper respiratory ways and skin). The use of hydroalcoholic extracts of propolis, although not chemically defined, was found to be effective in ameliorating the symptomatology in laryngitis, tonsillitis, and tracheitis [8]. Our group conducted a preliminary observational study [9] with 96 recruited subjects (36% males and 64% females, mean age 36.5 years old) to evaluate the efficacy of a commercial spray containing 316 mg/mL of propolis extract (45 mg/mL total flavonoids expressed as galangin) (*n* = 62) compared to flurbiprofen spray 2.5 mg/mL (*n* = 34) in the treatment of pharyngitis caused by common cold, non-infectious inflammation and streptococcal infections. Data showed that the symptoms (pain, difficulty in swallowing, cough) decreased in 85% of the subjects after using both propolis or flurbiprofen for 3 days, 3–4 times a day, with no side effects.

The aim of this research was to investigate pharmacological effects responsible for propolis effectiveness using different in vitro approaches for the same sample of European poplar type propolis (PP). The composition of the poplar propolis extract was chemically analyzed, as well as its anti-inflammatory activity, by determining pro-inflammatory cytokines (TNF-α, IL-1β, IL-2, IL-6, IL-17) produced by lipopolysaccharide (LPS)-stimulated human PBMC. Flurbiprofen (FLU) and CAPE were used as positive controls. The antibacterial activity of propolis against Gram+ and Gram- bacteria was assessed, as well as its antiviral effects on a H1N1 influenza A virus strain.

## 2. Materials and Methods

### 2.1. Propolis Sample

PP was furnished by Selerbe (Tavarnelle Val di Pesa, Firenze, Italy), with quality certificates for pesticides, antibiotics, and aflatoxins. PP was dissolved in ethanol 80% *v*/*v*, using an ultrasound bath for 2 h. The concentration of propolis in the ethanolic solution was adjusted to 400 mg.

### 2.2. Total Flavonoids Determination

Total flavonoids were analyzed according to Biagi et al. [10]. PP (400 mg/mL) was diluted 1:2000 in ethanol 80% *v*/*v* and total flavonoids were expressed as galangin by extrapolating the data in the calibration curve made up with galangin (reference standard grade, Sigma-Aldrich, Milan, Italy) 78–5000 mg/L, R^2^ = 0.987. Analyses were performed in triplicate.

### 2.3. HPLC-DAD Analysis

PP was analyzed by HPLC-DAD using a Shimadzu Prominence LC 2030 3D instrument equipped with a Bondapak^®^ C18 column, 10 µm, 125 Å, 3.9 mm × 300 mm column (Waters Corporation, Milford, MA, USA).

Water + 0.1% *v*/*v* formic acid (A) and methanol + 0.1% *v*/*v* formic acid (B) were used as mobile phases. The following method, according to Biagi et al. [11] with few modifications, was set: A: from 50% at 0 min to 30% after 12 min and then to 50% at 16 min; flow rate was set at 1.2 mL/min. Chromatograms were recorded at 280 nm.

Analyses were performed using 10 µL of PP solution in ethanol 80% *v*/*v* (400 mg/mL) diluted 100× and galangin, pinocembrin, and CAPE were used as external standards (Sigma-Aldrich, Milan, Italy). Calibration curves were establish using reference standards ranging from 0.008 to 0.500 mg/mL. The correlation coefficient (R^2^) of each curve was >0.99.

### 2.4. In Vitro Anti-Inflammatory Activity

PBMC were obtained by density gradient centrifugation [12]. After separation and isolation, PBMC (1 × 10^6^ cells/mL) were seeded in 24-well plates. PP was solubilized in ethanol 80% *v*/*v* (Sigma-Aldrich, Milan, Italy) and diluted in RPMI 1640 medium to obtain 1, 5, and 25 μg/mL in each well. CAPE and flurbiprofen (FLU) (Sigma-Aldrich, Milan, Italy) were solubilized in ethanol 80% *v*/*v* and used at the concentrations 0.1, 1, and 25 μg/mL. Cells were plated in duplicate and separated into non-stimulated and stimulated with LPS from *Salmonella enteridis* 200 ng/mL (Sigma-Aldrich, Milan, Italy). Dilution of ethanol 80% *v*/*v* present in 25 µg was used as solvent control. Negative controls, non-treated, and non-stimulated human PBMC, were also used. Three independent experiments were performed. All the plates were incubated for 24 h at 37 °C and 5% CO_2_.

Non-competitive sandwich ELISA kits (Biolegend e-Bioscience DX Diagnostic, Monza, Italy) were used to quantify TNF-α, IL-1β, IL-2, IL-6, and IL-17 in the supernatant of cell culture. Absorbances were recorded at 450 nm using a SAFAS MP96 spectrophotometer (Montecarlo, Monaco). Cytokine dosage was performed in duplicate for each sample.

### 2.5. Propolis Antibacterial Activity

The antibacterial activity of PP was investigated against *Staphylococcus aureus* (SA—CCUG 19207), methicillin-resistant *Staphylococcus aureus* (MRSA—CCUG 41879), *Staphylococcus epidermidis* (SE—CCUG 35257), *Streptococcus pyogenes* (SP—CCUG 12701), *Streptococcus pneumoniae* (SPN—CCUG 35180.), *Escherichia coli* (EC—CCUG 50175), and *Pseudomonas aeruginosa* (PA—CCUG 17619) (University of Göteborg collection).

Brain Heart Infusion Medium (BHI), Cation Adjusted Mueller Hinton Broth (CAMB) with 5% lysed equine blood, agar plates, and the multi-well plates were purchased from Biomerieux (Florence, Italy). PP (80% *v*/*v* ethanol) (250 mg/mL) was prepared by diluting the stock sample as reported in 2.1. The effects of PP solvent (ethanol 80% *v*/*v*) and of the positive control (amoxicillin—Sigma-Aldrich, Milan, Italy) were also analyzed.

The antibacterial activity was performed in a final volume of 100 μL in the culture medium [13]. In brief, serial dilutions of PP were made in the culture medium, the last column containing exclusively BHI (or CAMB with 5% lysed equine blood) for SP and SPN. In total, 2 μL of the different bacterial suspensions, containing approximately 5 × 10^6^ CFU, were added to each well. All experiments were performed in triplicate. The plates were incubated at 37 °C. After 24 h of incubation, the minimum inhibitory concentration (MIC) was determined as the minimum concentration leading to no visible bacterial growth, confirmed after inoculation of 2 μL of broth suspension in agar plates (Biomerieux, Florence, Italy) and incubation for 24 h at 37 °C.

### 2.6. Propolis Anti-Influenza Activity

The Puerto Rico swine influenza a virus A/PR/8 H1N1 was kindly furnished by Prof. A. Iorio, University of Perugia, Italy.

#### 2.6.1. Antiviral Activity (Pre-Infection)

Different dilutions of influenza virus (10–10^5^ TCID_50_ (median tissue culture infectious dose)/25 µL) were pre-incubated with 25 µL of PP solutions (3.13–100 µg/mL) for 30 min at room temperature. Afterwards, monolayer of Madin–Darby canine kydney cells (MDCK), cultured in DMEM (2 × 10^5^ cells/mL), were infected with 50 µL of different concentrations of virus treated with PP in a 96-well plate, then incubated at 35 °C and 5% CO_2_. The cytopathic effect (CPE) was observed daily using an optical microscope. Results were recorded after 4 days. The experiment was performed in four replicates. The concentration of PP capable of inhibiting the viral growth in all the replicates was considered as antiviral.

#### 2.6.2. Antiviral Activity (Post-Cell Absorption)

MDCK cells (2 × 10^5^/mL) were cultured in 96-well plates. The cells were infected with 200 TCID_50_ of A/PR/8 H1N1 influenza virus. After virus absorption for 1 h at 35 °C, the medium containing PP was added in each well and the plate was incubated at 35 °C and 5% CO_2_. CPE was observed daily and the results were read after 4 days. Oseltamivir (Sigma-Aldrich, Milan, Italy) was used as positive control (5 µg/mL in water). Also, this test was performed in four replicates. The concentration of PP capable of inhibiting the viral growth in all the replicates was considered as having antiviral activity.

#### 2.6.3. Cytotoxic Activity of PP on MDCK Cells

MDCK cells (2 × 10^5^/mL) were incubated with PP for 24 and 96 h. After, cells were stained with trypan blue (0.4%) to count dead cells. Each sample was analyzed in triplicate.

The percentage of cell viability after PP treatment was calculated by dividing the number of viable cells by the number of total cells after trypan blue staining.

#### 2.6.4. In Vitro Anti-Neuraminidase Activity

The evaluation in vitro of neuraminidase (NA) inhibition by PP was performed using a commercial kit (MAK-121 kit, Sigma Aldrich, Milan, Italy), according to the manufacturer’s procedures. Briefly, in a 96-multiwell plate in sextuplicate, 80 µL of the master reaction mix prepared with the reagents of the kit (buffer, enzyme, cofactors, and dye) were added to: (a) 20 µL of PP solutions, 4 to 0.063 mg/mL in ethanol 80% *v*/*v*, prepared by diluting the stock solution obtained as reported in 2.1; (b) 20 µL of oseltamivir, 0.25 to 0.008 mg/mL in distilled water, used as a reference drug; (c) 20 µL of assay buffer, in order to obtain 100% NA activity; (d) 20 µL of ethanol 80% *v*/*v* and water used as negative control. Finally, 20 µL of substrate standard (sialic acid) were added in each well. Plates were stirred in a horizontal shaker and incubated for 20 min at 37 °C and absorbance was read at 570 nm. The reading was repeated after a further 30 min. Absorbance recorded for each well after 50 min was subtracted from the absorbance recorded after 20 min. The percentage of inhibition of NA activity was calculated according to the formula:

%NA activity inhibition = 100 × (Abs_NAstand (c)_ − Abs_sample (a) or (b) or (d)_)/Abs_NAstand_. (1)

### 2.7. Statistical Analysis

Graphs and calculations were performed using GraphPrism^®^. The normality of the distribution was checked using D’Agostino and Pearson tests. Differences between the groups were determined by analysis of the variance (ANOVA), followed by Tuckey post hoc. Values were expressed as mean and standard deviation and *p* < 0.05 was considered statistically significant.

## 3. Results

### 3.1. Chemical Analysis of European Poplar Type Propolis

Total flavonoids expressed as galangin in PP (mg/g dry propolis) were 157.48 ± 15.02 mg/g.

HPLC analysis showed that the main flavonoid of PP was galangin, at a concentration of 42.25 mg/g dry material (retention time, RT, 12.87 min). A high concentration of pinocembrin was also seen: 27.30 mg/g dry propolis (RT = 10.61 min). By comparison with literature [11,14] and UV spectra, quercetin was also identified in the flavonoid pattern at RT 9.40 min. Flavonoid profile identified a typical and good quality poplar-type propolis, according to Gardana et al. [15], showing a higher content of total flavonoids in the range 14%–17% and galangin and pinocembrin as the most representative flavonoid markers.

Also CAPE (RT = 11.86) was found at high concentration, 11.21 mg/g of dry propolis, confirming the overall quality of the studied sample.

**Figure 1:**
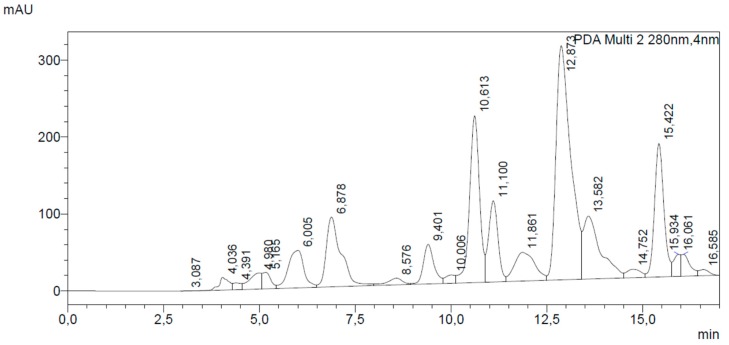
DAD chromatogram recorded at 280 nm of tested propolis sample. Galangin (RT = 12.87 min) and pinocembrin (RT = 10.61) were the main flavonoids in the extract.

Figure 1 and Table 1 show the chromatogram recorded at 280 nm and the quantification of the main analytes of the sample.

### 3.2. Anti-Inflammatory Effectiveness of Propolis Compared to CAPE and Flurbiprofen

LPS-stimulated human PBMC released the major pro-inflammatory cytokines: IL-1β was the most abundant cytokine compared to untreated cells. Also, IL-6 and TNF-α were highly upregulated. In contrast, LPS exerted no immunomodulatory action regarding IL-2 and IL-17 release. Table 2 shows cytokines production in LPS-stimulated human PBMC.

At basal conditions, PP (25 µg/mL) induced a slight upregulation in IL-1β (3.57 fold) and IL-6 (1.49 fold) production compared to control, and no effects were observed on TNF-α, IL-2, and IL-17. FLU 25 µg/mL increased IL-1β production (6.49 fold compared to control), while CAPE did not alter cytokine production by human PBMC. At lower concentrations, samples did not modulate cytokine release. Table 3 shows cytokine production in human PBMC treated with the samples 25 µg/mL.

**Figure 2:**
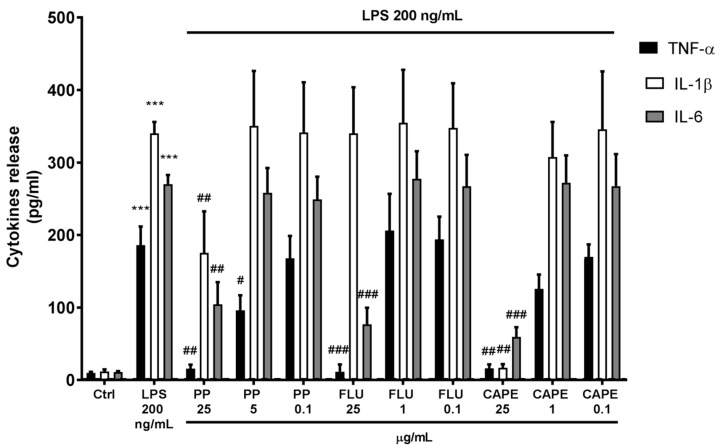
Effects of poplar propolis (PP), flurbiprofen (FLU), and CAPE on cytokine release by LPS-stimulated human PBMC. *** *p* < 0.05 vs. Ctrl; ^#^ *p* < 0.05 vs. LPS ^##^ *p* < 0.01 vs. LPS; ^###^ *p* < 0.001 vs. LPS.

As summarized in Figure 2, after LPS stimulation, PP exerted a concentration-dependent anti-inflammatory effect. Indeed, at 25 µg/mL, PP significantly reduced IL-1β (−48.46%), IL-6 (−61.42%), and markedly downregulated TNF-α (−91.65%) production, compared to LPS-stimulated human PBMC. Furthermore, at the concentration of 5 µg/mL, PP significantly reduced TNF-α by 48.93%, compared to LPS, while the concentration of 0.1 µg/mL was devoid of any effects. Similarly, CAPE (25 µg/mL) reduced IL-1β, IL-6, and TNF-α levels by 95.04%, 77.95%, and 91.22%, respectively. TNF-α seemed to be downregulated by CAPE 1 µg/mL (not in a statistical manner), but not at 0.1 µg/mL. CAPE 1 and 0.1 µg/mL exerted no effects on IL-6 and IL-1β. FLU 25 µg/mL significantly reduced IL-6 (−71.53%) and TNF-α (−93.87%) levels with an efficacy similar to that of PP at the same concentration; IL-1β seemed to be unaffected by FLU. Interestingly, no effects on cytokine production were observed using lower concentrations (1 and 0.1 µg/mL) of FLU.

### 3.3. Antibacterial Activity of Propolis Against Gram+ and Gram− Strains

Besides the anti-inflammatory capacity, other properties are ascribed to propolis, which could contribute to its clinical effectiveness in pharyngitis, sore throat, and common cold symptoms; first of all, anti-bacterial and antiviral characteristics. Thus, the antibacterial and anti-influenza activity was assessed herein. PP was much more active against Gram+ strains (except against *S. epidermidis*), compared to Gram- strains. Indeed, the MIC values against both *S. aureus* strains and *S. pneumoniae* were 625 µg/mL, while *S. pyogenes* was the most susceptible strain, with a very low MIC (156 µg/mL). One may verify that the antimicrobial effect of PP was not influenced by the solvent (i.e., ethanol), which exerted a dramatically higher MIC of 100,00–125,000 µg/mL against the tested strains. MICs obtained with PP are reported in Table 4.

### 3.4. Antiviral Activity of Propolis Against A/PR/8 H1N1 Influenza Virus

PP was not cytotoxic up to 25 µg/mL (87% of viable cells recorded at this concentration). Antiviral tests pre- and post-adsorption of H1N1 influenza virus strain on MDCK cells showed that PP, at non-cytotoxic concentrations, did not reduce the viral load. At higher concentrations, the antiviral activity corresponded to cytotoxicity, without a selective effect against H1N1. On the other hand, oseltamivir was effective and not cytotoxic at 5 µg/mL.

To better investigate the antiviral activity of PP, overcoming a possible cytotoxicity issue on MDCK cells, an in vitro enzymatic test was performed evaluating the inhibition of neuraminidase (NA) activity. The test proved to be reliable, as confirmed by the IC_50_ of oseltamivir. PP showed an interesting low value of IC_50_, 35.29 µg/mL, similar to other effective herbal extracts such as *Salix* spp. or *Nelia meyeri* Schwantes, recently tested by Quosdorf et al. [16]. IC_50_ value of PP is also consistent with a concentration higher than the non-cytotoxic one. The IC_50_ of both oseltamivir and PP are shown in Table 5.

## 4. Discussion

Bee propolis is among the most used natural products worldwide, especially to treat sore throat, skin wounds, and mouth disorders [7]. Nevertheless, in Western countries physicians scarcely consider the use of propolis in conventional medicine. Two important pitfalls may principally occur: few clinical studies, which fail to precisely address medical indications of propolis and the large variability in chemical composition of propolis of different origins [17].

Regarding the latter point, we previously reported that the best studied propolis, the Euro-Asian poplar type (collected by bees prevalently from *Populus* spp.), exert biological effect due to its peculiar polyphenolic composition (flavonoids in particular) that contributes for antioxidant, immunomodulatory, and antimicrobial effects [7,11,18]. Thus, we can state that only a chemically characterized poplar propolis could be used for medical purposes and polyphenolic profile should be always analyzed.

This work aimed to better investigate different putative biological characteristics, namely antibacterial, antiviral, and anti-inflammatory activity, of a poplar propolis (PP) sample analyzed for galangin, pinocembrin, and CAPE content. Indeed, pharyngitis presents typical inflammatory symptoms such as burning and reddening, but they are often linked to viral and, in a lesser extent, to bacterial infections, especially caused by *Streptococcus* spp. [19].

Overall, PP was effective in counteracting cytokines release in inflammatory conditions, using LPS-stimulated human PBMC. As reported by De Groote and colleagues [20], we found that bacterial LPS induced a typical monocytes response in human PBMC, characterized by TLR4/NF-κB-driven upregulation of IL-1β, IL-6, and TNF-α. CAPE has been reported as one of the most active anti-inflammatory constituents of PP-targeting NF-κB [21]. Thus, its activity was compared to whole PP. CAPE (25 µg) exerted the highest inhibitory effect on cytokines release by LPS-stimulated human PBMC. Besides, at 1 µg/mL, CAPE partially downregulated TNF-α release and was ineffective at lower concentrations. Considering that CAPE was found to count for 1% ca. in PP, at the studied concentration (25 µg/mL) PP only contains 0.25 µg/mL ca. of CAPE, suggesting that the anti-inflammatory efficacy of PP is not only dependent on CAPE presence. As observed in many other natural products, the whole propolis phytocomplex seems to be responsible for observed biological effects. Reactive radical species play a pivotal role in NF-κB activation [22], thus, we can suggest the antioxidant and antiradical capacities of the flavonoid pool of PP to participate in its effectiveness.

The anti-inflammatory effect of PP was compared to FLU and PP (25 µg) exerted a similar activity to the reference drug at the same concentration in inhibiting IL-6 and TNF-α release in LPS-stimulated human PBMC and a better efficacy in inhibiting IL-1β.

Interestingly, PP was found to possess additional biological characteristics, exploitable for its medical use. Indeed, PP was shown to slightly upregulate IL-6 and IL-1β in non-stimulated human PBMC. The ability of propolis to upregulate immune cell activation has been clearly reported using Brazilian green propolis [23] and its immunomodulatory activity is a relevant characteristic of this bee product, pointing out its contribution in clinical assays [24].

A direct antiviral activity against influenza virus A H1N1 was not clearly demonstrated due to a low ratio between inhibitory concentration of cytopathic effect and cytotoxic concentration. However, a plausible role of PP on virus entry and replication in host cells, dependent on anti-NA activity, was, at least in part, confirmed by the in vitro enzymatic test. NA, together with hemoagglutinin, dominated the outer surface of viral structure both in influenza virus A and B. NA involves interaction with sialic acid, bound to sugar residues of glycoproteins at the cell surface. NA cleavage of sialic acids drives efficient release of virus to new host targets, but it also exerts a role in viral cell entry [25]. The development of new anti-influenza drugs is still focusing on NA, which is the target of oseltamivir and zanamivir. Currently, these antiviral agents have a limited clinical use since the risk–benefit ratio is seriously debated worldwide [26]. The inhibition of other fundamental viral enzymatic targets exerted by CAPE, such as HIV-1 integrase, has been reported [27].

In this work, we also confirmed the efficient antibacterial activity of PP and *S. aureus*, *S. pyogenes*, and *S. pneumoniae* were the most susceptible strains. As previously reported, galangin and pinocembrin may be involved in the antibacterial activity of propolis, increasing the bacterial membrane permeability [28] and inhibiting RNA polymerase [29]. These flavonoids, found in high concentration in PP, plausibly contribute to the effectiveness of PP. In the strenuous attempt of overcoming microbial resistance, this bee product should be considered and extensively used for external and mucosal infections when conventional antibiotics are not strictly recommended. In fact, similar MIC values for methicillin-resistant and not resistant *S. aureus* and higher activity against Gram+ bacterial strains, compared to Gram- strains, were recorded, suggesting that propolis may primarily trigger bacterial membrane permeability in a physical manner [28]. Moreover, the mixture of all the “phytoactive” components of propolis, as proposed and widely discussed for many other natural products, may exert a multitarget effects, which could slow down microbial resistance [29,30].

## 5. Conclusions

Different pharmacological properties were investigated, indicating the effectiveness of PP as an anti-inflammatory, immunomodulatory, and antimicrobial agent.

It is worthy to note that PP concentrations able to exert anti-inflammatory activity were significantly lower than those able to exhibit antibacterial and antiviral activities. Our data suggest the anti-inflammatory efficacy as the most important feature of PP in pathological conditions such as sore throats and upper respiratory diseases.

The inhibition of cytokines released by human PBMC was clearly demonstrated at low concentrations, which were similar to FLU and available for local treatment with oral spray or other topical preparations.

## Figures and Tables

**Table 1:** Content of the main chemical markers of the studied poplar-type propolis (PP).

Constituent	Content [mg/g Dry Propolis]
galangin	42.25 ± 0.51
pinocembrin	27.30 ± 0.26
CAPE	11.21 ± 0.10

**Table 2:** Cytokines release by LPS-stimulated human PBMC. *** *p* < 0.001 vs. ctrl.

Parameter	Control	LPS
	Cytokines dosage (pg/mL)	
TNF-α	9.76 ± 1.60	185.95 ± 25.78 ***
IL-1β	11.77 ± 2.91	340.01 ± 15.94 ***
IL-2	4.24 ± 0.57	4.50 ± 1.01
IL-6	10.84 ± 1.48	270.31 ± 12.64 ***
IL-17	17.10 ± 3.45	16.44 ± 3.65

**Table 3:** Cytokines release by human PBMC treated for 24 h with poplar propolis (PP), flurbiprofen (FLU), and CAPE (25 µg/mL). * *p* < 0.05 vs. ctrl; ** *p* < 0.01 vs. ctrl.

Sample		Parameter				
	**Concentration (** **μg/mL)**	**TNF-** **α**	**IL-1** **β**	**IL-2**	**IL-6**	**IL-17**
		**Cytokines Dosage (pg/mL)**				
PP	25	10.83 ± 1.44	42.02 ± 8.17 *	4.20 ± 0.34	16.15 ± 1.21 *	15.05 ± 1.26
FLU	25	11.71 ± 1.19	76.39 ± 16.00 **	4.32 ± 0.48	12.47 ± 0.95	16.76 ± 1.58
CAPE	25	9.86 ± 0.98	12.95 ± 1.29	3.90 ± 0.32	10.95 ± 0.99	15.39 ± 1.44

**Table 4:** Minimum inhibitory concentrations (MICs) of PP against different Gram+ and Gram− bacterial strains.

Bacterial Strain	MIC (μg/mL)
*S. aureus*	625
MRSA	625
*S. epidermidis*	2500
*S. pyogenes*	156
*S. pneumoniae*	625
*E. coli*	5000
*P. aeruginosa*	2500

**Table 5:** Anti-neuraminidase activity of PP and oseltamivir.

Sample	IC_50_ (μg/mL)
oseltamivir	5.88 ± 0.89
PP	35.29 ± 4.08
